# Development of a tunable method to generate various three-dimensional microstructures by replenishing macromolecules such as extracellular matrix components and polysaccharides

**DOI:** 10.1038/s41598-020-63621-4

**Published:** 2020-04-16

**Authors:** Fumiya Tao, Kanae Sayo, Kazuyuki Sugimoto, Shigehisa Aoki, Nobuhiko Kojima

**Affiliations:** 1 Graduate School of Nanobioscience, Yokohama City University, Yokohama, Japan; 2 Solution Division, Quality Assurance and Customer Support Center, Life Innovation Business Headquarters, Yokogawa Electric Corporation, Kanazawa, Japan; 3 Department of Pathology & Microbiology, Faculty of Medicine, Saga University, Saga, Japan

**Keywords:** Cell culture, Tissue engineering

## Abstract

Multicellular spheroids (spheroids) are expected to be a promising approach to mimic *in vivo* organ functions and cell microenvironments. However, conventional spheroids do not fully consider the existence of extracellular matrices (ECMs). In this study, we developed a tunable method for replenishing macromolecules, including ECM components and polysaccharides, into spheroids without compromising cell viability by injecting a microvolume cell suspension into a high density of methylcellulose dissolved in the culture medium. Adjusting the ECM concentration in the cell suspension enabled the generation of different three-dimensional microstructures, such as “ECM gel capsules”, which contained individually separated cells, and “ECM-loaded spheroids”, which had thin ECM layers between cells. ECM-loaded spheroids with a 30-fold dilution of Matrigel (0.3 mg/ml) showed significantly higher albumin secretion than control spheroids composed of Hep G2 or HuH-7 cells. Additionally, the expression levels of major CYP genes were decreased in ECM gel capsules with undiluted Matrigel (9 mg/ml) compared to those in control spheroids. However, 0.3 mg/ml Matrigel did not disrupt gene expression. Furthermore, cell polarity associated with tight junction proteins (ZO-1 and Claudin-1) and the transporter protein MRP2 was markedly induced by using 0.3 mg/ml Matrigel. Thus, high-performance three-dimensional tissues fabricated by this method are applicable to increasing the efficiency of drug screening and to regenerative medicine.

## Introduction

Spheroid culture is one of the three-dimensional (3D) cell culture methods bridging gaps between *in vitro* monolayer cultures and *in vivo* tissue functions that are modulated by cell-cell and cell-extracellular matrix (ECM) interactions. For example, spheroids composed of hepatocytes produce more tissue-specific molecules, albumin and urea and exhibit higher levels of metabolic functions, including drug metabolism, than cells in monolayer culture^1–4^. Laschke *et al*.^5^ reviewed various methods to assemble spheroids and discussed their advantages. For example, spheroids of the hepatoma cell lines Hep G2 and HuH-7 were successfully generated in hanging drops or on low adhesion surfaces^6–10^. However, these culture systems do not fully consider the influence of the ECM. The composition and distribution of the ECM differs among organs and tissues *in vivo*, thereby affecting cell survival, morphology, polarity, and differentiation^11–13^. In addition, the ECM is pathologically altered either as a cause or as a result of multiple disease processes^14^. Therefore, the ECM is one of the key factors to be considered for tissue engineering applications.

A 3D culture method that allows modulation of the ECM amount (thickness) between cells is critical for the fabrication of normal and diseased tissues in order to reflect the structure and function of tissues *in vivo*. Several techniques have been proposed to control the ECM thickness between cells in spheroids. For example, a layer-by-layer cell coating technique has been developed that enables coating of the cell surface with ECM-derived components, namely, fibronectin and gelatin^15^. A method of generating micrometre-sized ECM scaffolds on cell surfaces via the formation of type I collagen fibres has also been reported^16^. Although these techniques can precisely control the thickness of the ECM between cells, multi-step manipulation is required to fabricate a thick ECM layer. Furthermore, the available types of ECMs are restricted, because this technique depends on the interaction between two types of ECM components or between integrin receptors and ECM components. Another approach is encapsulating cells into an ECM gel by water-in-oil emulsion methods^17,18^. This approach allows the fabrication of 3D tissues with a thick ECM layer between cells in one step and can also replenish thin ECM layers between cells by increasing the initial cell density per capsule. However, the usage of oil could significantly decrease the viability of cells encapsulated in ECM gels, especially in capsules with a high cell density, because the mineral oil may also have adverse effects due to the possibility that cell toxic contaminations such as aromatic and unsaturated hydrocarbons that accumulate in the oil during production, transport or storage^19,20^. Thus, it is necessary to develop a novel 3D culture method that allows (1) single-step, (2) successive regulation of the ECM amount between cells, (3) utilization of various ECM components, and (4) prevention of cell death.

Previously, we established a method to generate spheroids using a high molecular weight material medium: methylcellulose (MC) medium^21^. It has been reported that ECMs are incorporated into spheroids using MC as catalysis^22,23^. These methods utilize MC as a catalyst for generating spheroids. However, the aggregation efficiency of cells is different depending on the cell type. In contrast, our method to assemble spheroids utilizes the absorbing and swelling property of a solution containing a high concentration of MC from liquid in an injected cell suspension. The method can generate a 3D aggregate state regardless of the adhesive properties of the cells (e.g., bone marrow cells)^24^. In particular, the aggregation method using MC medium allowed us to accumulate living cells and polystyrene particles of 0.1 µm to 100 µm in diameter^21,25^. The ECM molecules type I collagen-conjugated FITC and type IV collagen are 0.3 µm and 0.4 µm in length, respectively^26,27^. Laminin, one of the components of the ECM basement membrane, forms complexes with type IV collagen and has a cruciform structure with two short arms (0.034 and 0.042 µm in length) and a single long arm (average length of 0.097 µm)^28^. Therefore, we hypothesized that the MC medium method can aggregate cells and ECM molecules simultaneously.

In this study, we present a 3D culture method that can successively tune the amount of several ECMs between cells in spheroids without compromising the cell viability. Furthermore, the effect of these ECMs on cellular functions and cell polarity was demonstrated in hepatic spheroids.

## Results

### Co-aggregation and the resulting distribution of cells and macromolecular proteins in MC medium

**Figure 1:**
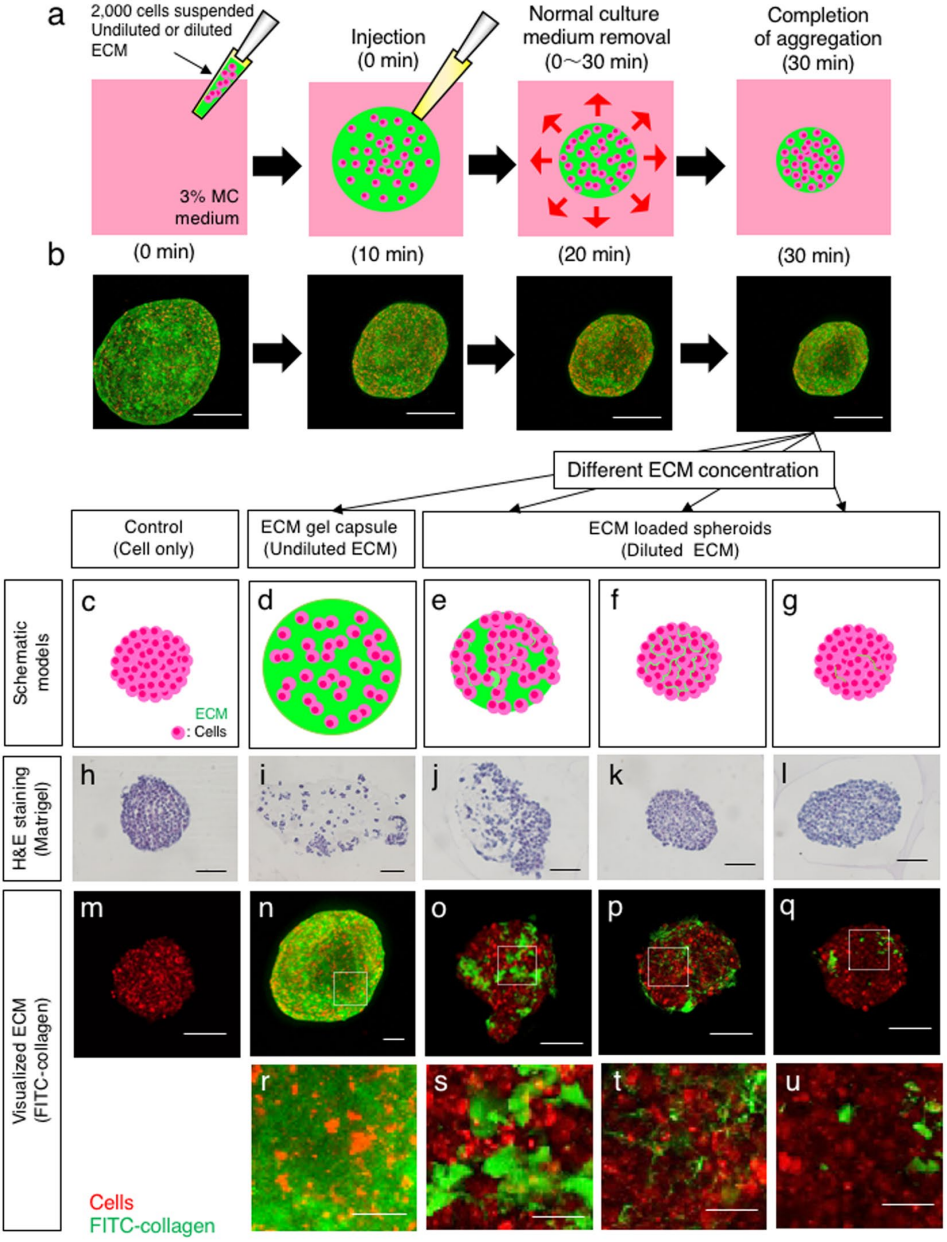
Co-aggregation of cells and macromolecules with high and low concentrations in MC medium. (**a**) Schematic overview of aggregation with macromolecules such as ECM components and polysaccharides in MC medium. (**b**) Injected suspension mixture of PKH26-stained Hep G2 cells (red) with FITC-collagen (green) at the indicated time points after injection. Bar = 200 µm. (**c–g**) Schematic overview of 3D tissues composed of cells and different concentrations of ECM. (**h–l**) Haematoxylin and eosin staining of 3D tissues loaded with the indicated concentrations of Matrigel. Bar = 100 µm. (**m–q**) ECM in the spheroids was visualized by using different dilutions of FITC-collagen (green) together with Hep G2 cells (red) as in (**b**). Bar = 100 µm. (**r–u**) are the insets in (**n–q**) surrounded by dashed line rectangles. Bar = 20 µm.

As we previously reported, 3% MC medium is suitable for the rapid aggregation of mammalian cell suspensions mixed with non-adhesive artificial particles^21,25^. To examine whether biological ECM components can co-aggregate with cells, cells labelled with PKH26 were re-suspended with FITC-collagen and injected into MC medium, and the progression of aggregation was observed over time (Fig. 1a). The red-labelled cells co-aggregated with green-labelled collagen over time, forming a 3D tissue containing ECM and a uniform distribution of cells (Fig. 1b). The high concentration of ECM was expected to form a gel capsule as individual cells were surrounded by ECM. To investigate whether cell capsulation and the resulting inhibition of cell-cell interaction was ECM concentration-dependent, 3D tissues were prepared by using various concentrations of Matrigel and were subjected to paraffin sectioning and haematoxylin-eosin staining (Fig. 1h–l). Compared with the ECM gel capsule (undiluted Matrigel) (Fig. 1i), in which cells were distributed separately without forming large masses, the cells in spheroids with a 15-fold dilution of Matrigel (0.6 mg/ml) (Fig. 1j) aggregated, although the cells were not in full contact, unlike the no-ECM control spheroids (Fig. 1h). Under 30- and 100-fold dilution conditions (0.3 mg/ml and 0.09 mg/ml, respectively; Fig. 1k,l), the structures of spheroids were similar to those of no-ECM control spheroids, without thick ECM layers. We also observed the ECM distribution in the spheroids after one day of culture, as visualized by collagen-conjugated FITC. As the concentration of FITC-collagen was decreased, the thickness of the ECM gel in the gaps between cells decreased, and the cell-cell interaction increased in spheroids (Fig. 1n–q). At a FITC-collagen concentration of 0.1 mg/ml, a thin film-like layer formed between cells (Fig. 1p,t), although it was not identified by haematoxylin and eosin staining. That structure was barely observed in spheroids with a lower FITC-collagen concentration (0.03 mg/ml, Fig. 1q,u). However, a higher ECM concentration (0.2 mg/ml, Fig. 1o,s) resulted in the formation of cell masses dispersed in spheroids. When we used 0.2 mg/ml, 0.1 mg/ml or 0.03 mg/ml FITC-collagen, the thickness of ECM gel between cells in spheroid were quantified as approximately 1.264 µm, 0.857 µm and 0.741 µm respectively (see Supplementary Fig. S1). The amount of ECM per spheroid was also quantified by using various concentrations of FITC-labelled ECM. When we used 3 mg/ml, 0.2 mg/ml, 0.1 mg/ml or 0.03 mg/ml FITC-collagen, the percentages of ECM amounts per spheroid were quantified as approximately 95%, 26%, 13% and 2%, respectively (see Supplementary Fig. S1). In addition, the amounts of FITC-labelled fibronectin per spheroid using 0.5 mg/ml, 0.1 mg/ml or 0.01 mg/ml were 7%, 3% and 0.6%, respectively (see Supplementary Fig. S1). We defined such spheroids with diluted ECM as “ECM-loaded spheroids” to distinguish them from the “ECM gel capsules”. These results indicate that the structures of 3D spheroid tissue at the endpoint of aggregation vary depending on the concentrations of ECM.

### 3D structures corresponding to ECM concentration and temperature

**Figure 2:**
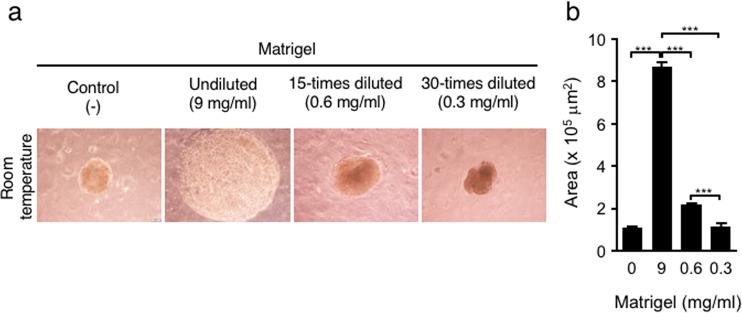
Sizes of Hep G2 spheroids in MC medium corresponding to the Matrigel concentration. Injection of Matrigel and cells was performed at room temperature and cultured for one day (**a**). Images used to measure spheroid areas (**b**). The data are the means ± SEMs, n = 7–10. *p  <  0.05, **p  <  0.01, ***p  <  0.001 by two-way ANOVA.

The role of ECM in the progression of spheroid aggregation was examined by using various concentrations of Matrigel, followed by calculation of the average area of the spheroid cross sections one day after injection. The samples formed from ECM gel capsules with undiluted Matrigel (9 mg/ml) had a greater expanded area than control spheroids (8.6 × 10^5^ µm^2^ vs. 1.1 × 10^5^ μm^2^) (Fig. 2). The area of spheroids decreased significantly in response to Matrigel dilution, quantified as 2.1× 10^5^ μm^2^ with 0.6 mg/ml Matrigel and 1.1 × 10^5^ μm^2^ with 0.3 mg/ml Matrigel. The area of spheroids with 0.3 mg/ml Matrigel was almost the same as the area of control spheroids. Although those spheroids were produced at room temperature and incubated at 37 °C, temperature is another factor influencing ECM gelation. Matrigel is a liquid below 8 °C and initiates gelation between 22 °C and 37 °C^29^. To investigate the influence of ECM gelation, which can be prevented at low temperatures, all the procedures—mixing the cell suspension and Matrigel, injecting the suspension into MC medium, and incubating for 60 minutes—were performed at 4 °C. After spheroids were cultured for one day at 37 °C, we measured the cross-sectional area of the spheroids (Fig. 2 and see Supplementary Fig. S3). When we used undiluted (9 mg/ml) or a 15-fold diluted (0.6 mg/ml) Matrigel, the areas of the spheroids were significantly smaller than the areas of spheroids fabricated at room temperature. In addition, the areas of control spheroids and ECM-loaded spheroids using a 30-fold dilution of Matrigel (0.3 mg/ml) were found to be the same between 4 °C and room temperature conditions (Fig. 2 and see Supplementary Fig. S4). These results imply that a high concentration of ECM suppresses the progression of aggregation to form spheroids at higher temperatures, which inhibits ECM condensation during aggregation by gelation.

### Molecular weight-dependent aggregation and diffusion of polysaccharides after injection into MC medium

**Figure 3:**
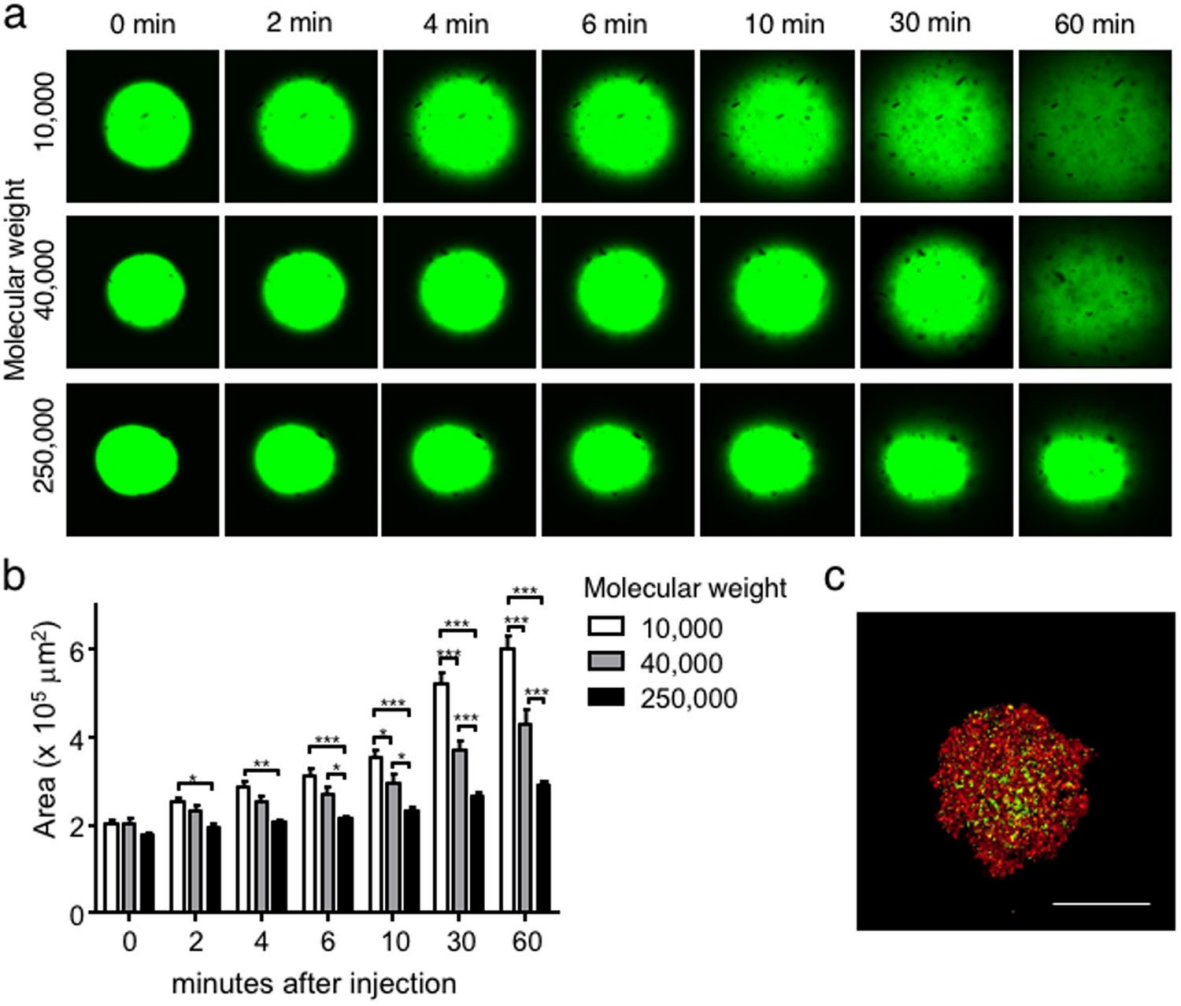
Aggregation with different molecular weights of polysaccharides in MC medium. (**a**) Three different molecular weights of FITC-dextran with average sizes of 10,000, 40,000 and 250,000 were injected into MC medium. The time-dependent dextran distribution was observed (**a**), and fluorescent areas were quantified (**b**). Bar = 200 µm. The data are the means ± SEMs, n = 3. *p  <  0.05, **p  <  0.01, ***p  <  0.001 by two-way ANOVA vs. the 10,000 group at each time point. (**c**) Hep G2 cells labelled with PKH26 (red) and molecular weight of 250 FITC-dextran (green) were mixed and injected into MC medium. After one day of culture, aggregation was observed. Bar = 200 µm.

To investigate the molecular size that can be retained in MC medium after condensation, FITC-dextran solution with average molecular weights of 10,000, 40,000, and 250,000 was injected. Whereas FITC-dextran with molecular weights of 10,000 and 40,000 diffused in MC medium until 60 minutes after injection, FITC-dextran with molecular weight of 250,000 did not diffuse in MC medium (Fig. 3a). When the distribution (diffusion) areas of FITC-dextran were quantified, the areas of FITC-dextran with molecular weights of 10,000 and 40,000 were significantly larger than those of FITC-dextran with molecular weight of 250,000 at two minutes and six minutes after injection, respectively (Fig. 3b). Then, the differences were increased at later time points by progressive diffusion of FITC-dextran with smaller molecular weights. These results indicate that MC medium has the potential to prevent the diffusion of macromolecules such as molecular weight of 250,000. When a cell suspension mixed with 1.25 mg/ml molecular weight of 250,000 FITC-dextran was injected into MC medium, FITC-dextran filled the gaps between the cells in spheroids and the ECM without diffusion, as well as within the ECM (Fig. 3c). The molecular weights of type I collagen, fibronectin, and laminin comprising the ECMs in the tissues were 300,000, 450,000–500,000, and 800,000, respectively^30–32^. Hence, any water-soluble macromolecules may exhibit similar behaviours of aggregation and replenishment between cells without diffusion in MC medium.

### Reduced cytotoxicity during encapsulation by using MC medium compared with conventional methods

**Figure 4:**
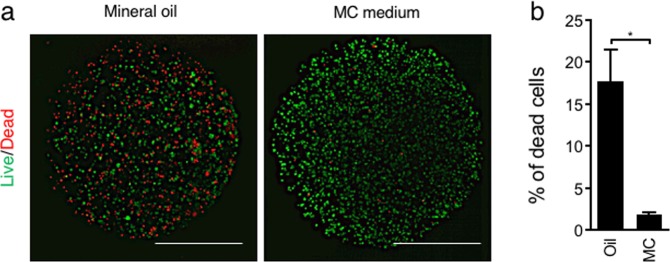
Viability of encapsulated Hep G2 cells aggregated by conventional oil and MC medium methods. (**a**) ECM gel capsules were stained with propidium iodide (red) to identify dead cells and calcein-AM (green) to identify living cells. (**b**) The percentage of dead cells in ECM capsules was calculated. ECM capsules treated with 4% paraformaldehyde were used as 100% dead spheroid controls. Bar = 500 µm. The data are the means ± SEMs, n = 3. *p  <  0.05 by Student’s t-test.

One of the typical 3D culture methods to replenish the ECM is to embed cells in the capsule of an ECM gel, which is formed in an emulsion containing aqueous drops consisting of cells and ECM in oil. In this method, some cells are damaged due to contact with oil. Accordingly, cytotoxicity was investigated when Hep G2 cells were encapsulated in ECM gel formed in conventional oil emulsion and formed in MC medium, which does not use oil. Cell viability was examined by using LIVE/DEAD reagent, which demonstrated a marked red-stained dead cell population in ECM gel capsules formed in mineral oil, whereas few dead cells were observed in ECM gel capsules formed in MC medium (Fig. 4a). As a result of quantitative evaluation, the population of dead cells was significantly higher in the samples using oil (17%) than in those in MC medium (1.8%) (Fig. 4b). Mineral oil, as used for mouse embryo culture, does not contain any cytotoxic compounds^33^. However, cytotoxicity was observed specifically with mineral oil, suggesting that cell viability is decreased by any operation that brings cells into contact with oil.

### Effect of loading a thin ECM layer into spheroids on albumin secretion

**Figure 5:**
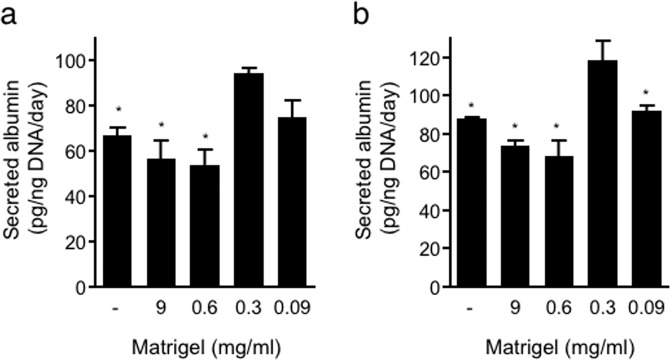
Albumin secretion from Hep G2 and HuH-7 spheroids in response to various Matrigel concentrations. Secreted albumin levels in culture medium were measured by ELISA. In addition, spheroids incubated for albumin secretion assessment were extracted, and the amount of genomic DNA was measured to normalize albumin secretion by the number of Hep G2 (**a**) and HuH-7 (**b**) cells. The data are the means ± SEMs, n = 3. *p  <  0.05 by one-way ANOVA vs. data obtained with 0.3 mg/ml Matrigel.

Albumin secretion is one of the major indicators of hepatocyte function. To investigate the effect of the ECM and its concentration dependency on albumin secretion, Hep G2 or HuH-7 spheroids fabricated with a series of Matrigel dilutions were cultured for 24 hours, and conditioned medium was then collected to measure the level of secreted albumin. Spheroids with a high concentration of Matrigel (9 and 0.6 mg/ml) had the lowest albumin secretion rates. However, ECM-loaded spheroids with a lower concentration of Matrigel (0.3 mg/ml) secreted more albumin than no-ECM control spheroids (Fig. 5). However, further Matrigel dilution (0.09 mg/ml) did not show such an increase in albumin secretion. This finding implies an optimal Matrigel concentration to achieve an ECM-loaded microenvironment in spheroids for albumin secretion. This finding also implies that too much ECM will suppress hepatic function, probably because the excess ECM inhibits cell-cell interactions, as shown in Fig. 1. To investigate long-term stability of hepatic function, smaller spheroids composed of 1,000 cells were generated and cultured until 7 days. All culture conditions (with a series of Matrigel or without) were shown similar amount of albumin secretion at day 7 of culture(see Supplementary Fig. S4).

### Liver-specific gene expression in spheroids in response to the ECM concentration

**Figure 6:**
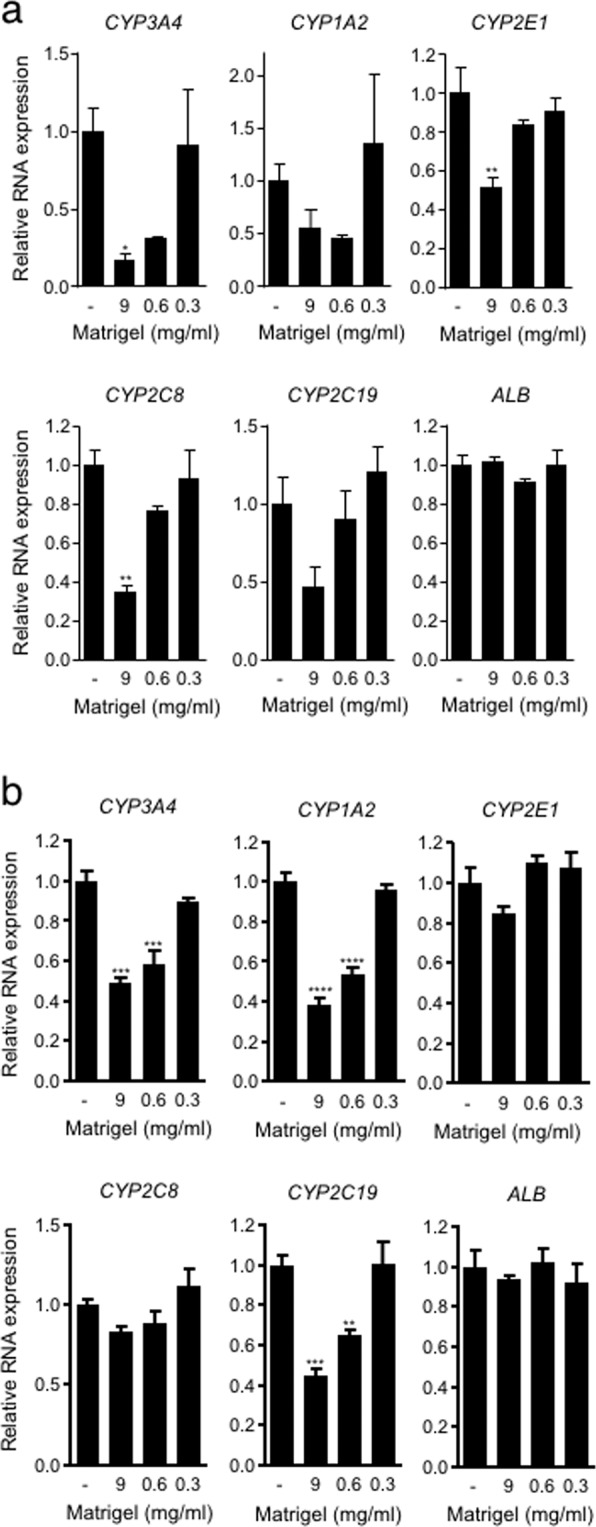
Gene expression analyses for CYPs and albumin in spheroids using Hep G2 cells and HuH-7 cells two days after injection. Data are shown as the fold change against expression level in no-ECM control spheroid consisting of Hep G2 (**a**) and HuH-7 (**b**). The data represent the mean ± SEM, n = 3. *p  <  0.05. **p  <  0.01. ***p  <  0.001 by one-way ANOVA vs. no-ECM control.

One of the major functions of the liver is the metabolism of drugs and toxins, and occasional drug-drug interactions mediated especially by cytochrome P450 (CYP) enzymes may occur^34^. Thus, the effect of various Matrigel concentrations on CYP gene expression was examined by real-time PCR. The expression levels of several CYPs by cells in ECM gel capsules with undiluted Matrigel (9 mg/ml) were decreased compared to those in control spheroids, although the response of CYP species was largely different between Hep G2 and HuH-7 cells. In Hep G2 cells, the expression levels of CYP3A4, 2E1 and 2C8 were lower in ECM gel capsules than in no-ECM control spheroids (by 17%, 51%, and 35%, respectively) (Fig. 6a). In HuH-7, the expression levels of CYP3A4, 1A2, and 2C19 were significantly decreased (by 49%, 38%, and 44%, respectively) in ECM gel capsules compared to those in control spheroids (Fig. 6b). As the concentration of Matrigel was decreased, the expression levels of CYPs gradually increased. Under 30-fold dilution conditions (0.3 mg/ml Matrigel), gene expression became similar to that in control spheroids in both Hep G2 and HuH-7 cells. Subsequently, the gene expression of albumin was also assessed. In contrast to the result of the albumin secretion assay, albumin gene expression remained almost unchanged under all conditions (Fig. 6a,b). The expression of these genes was also studied to evaluate long-term stability as with the albumin secretion assay. Spheroids with a series of Matrigel dilutions were shown similar level of gene expression except for CYP1A2 as no-ECM control spheroids at day 7 of culture (see Supplementary Fig. S4). These results indicate that it is possible to replenish the ECM in spheroids without inhibiting liver-specific gene expression by diluting the ECM.

### Localization of cell polarity-related proteins in spheroids in the presence or absence of ECM

**Figure 7:**
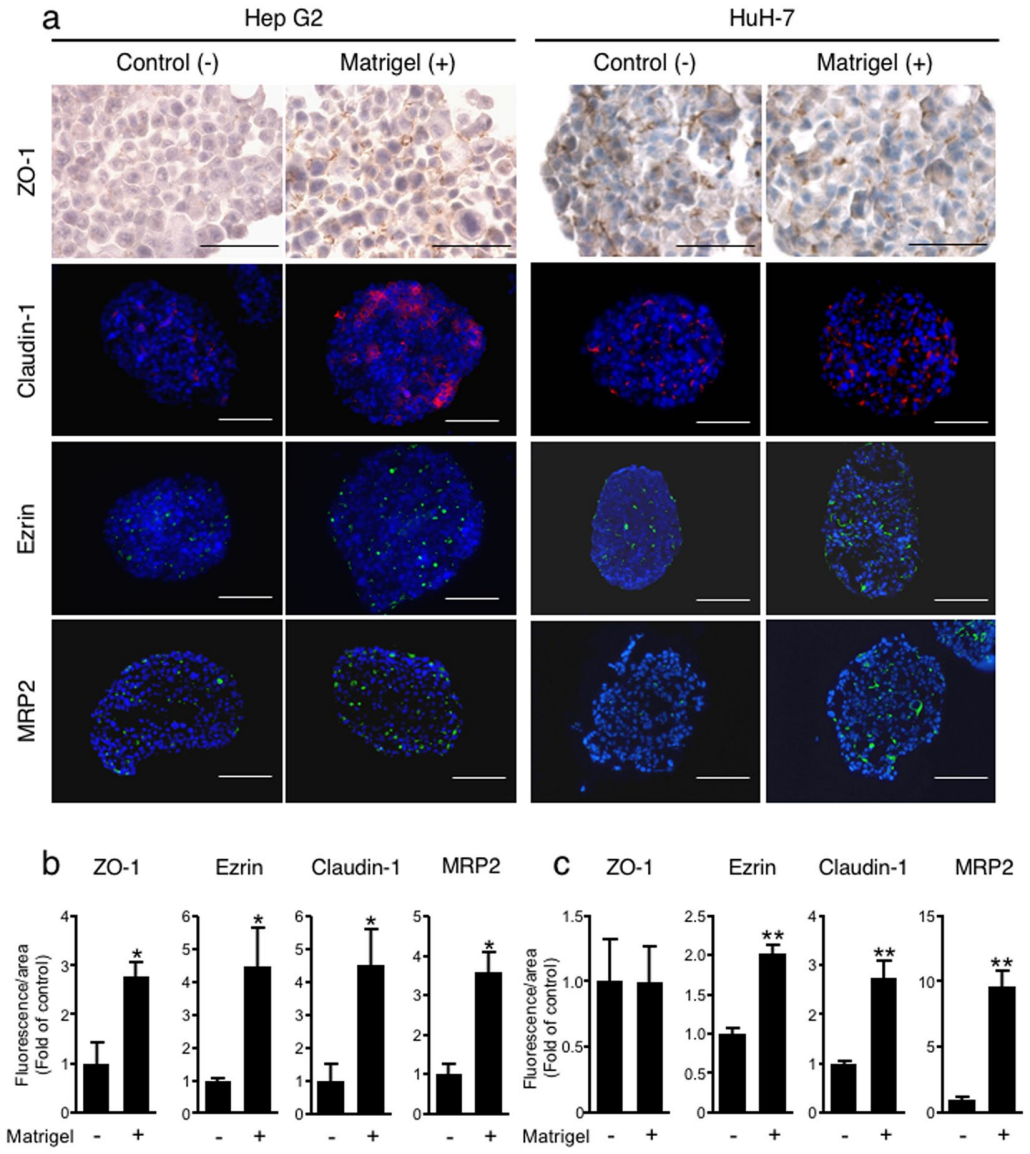
Immunostaining of cell polarity-related proteins. (**a**) Control spheroids and ECM-loaded spheroids with 0.3 mg/ml Matrigel were fixed two days after injection into MC medium, followed by sectioning and staining for the tight junction-associated proteins ZO-1 (DAB staining) and Claudin-1, the apical marker Ezrin, the bile acid transporter MRP2 (red fluorescence) and staining with Hoechst 33342 for nuclei (blue). Bar = 50 µm. Positive areas for ZO-1, Claudin-1, Ezrin, and MRP2 were quantified and normalized to the nuclear area of Hep G2 (**b**) and HuH-7 (**c**) spheroids. The data are shown as the fold of expression in control spheroids. The data are the means ± SEMs, n = 3–4. *p  <  0.05. **p  <  0.01 by Student t-test.

Matrigel is a mixture of ECM components, including basement membrane components such as laminin, type IV collagen and fibronectin, which are related to cell polarity acquisition. To study the effect of Matrigel loading (0.3 mg/ml) into spheroids on cell polarity formation, ZO-1 and Claudin-1, which are associated with tight junctions, were identified by immunostaining in Hep G2 and HuH-7 spheroids. In Hep G2 spheroids, a marked increase in ZO-1 and Claudin-1 signals was observed in Matrigel-loaded spheroids. However, HuH-7 spheroids exhibited no difference in the localization of ZO-1 and Claudin-1 regardless of the presence or absence of Matrigel. The expression of Ezrin, an apical protein in liver tissue *in vivo*, was clearly induced in Matrigel-loaded spheroids of both Hep G2 cells and HuH-7 cells. These results suggest that Matrigel-loaded spheroids develop tight junctions and cell polarization. MRP2 is a transporter that secretes bile from the apical side of hepatocytes; therefore, its localization was also examined by immunostaining. Both Hep G2 cells and HuH-7 cells exhibited induction of MRP2 expression in Matrigel-loaded spheroids. Areas positive for ZO-1, Claudin-1, Ezrin and MRP2 signals were quantified and normalized to the nuclear area in each section. ECM-loaded spheroids with 0.3 mg/ml Matrigel formed from both Hep G2 and HuH-7 cells exhibited significantly increases in the relative amounts of ZO-1, Ezrin and MRP2 compared to those in control spheroids, except for ZO-1 in HuH-7 spheroids (Fig. 7b,c). MRP2 is a transporter localized on the bile canalicular membrane of the apical side of hepatocytes and functions to export bile acid from the cell to the bile canaliculi. We investigated whether bile canaliculi-like structures were formed in ECM-loaded spheroids containing Matrigel. Compared to control spheroids, ECM-loaded spheroids of both Hep G2 and HuH-7 cells with 0.3 mg/ml Matrigel exhibited an easily observable bile canaliculus with microvilli (see Supplementary Fig. S5).

## Discussion

In this study, we developed a novel 3D culture method that fulfilled the requirements mentioned above: (1) single-step; (2) successive regulation of the ECM amount between cells; (3) utilization of various ECM components; and (4) maintenance of cell viability.

We found that the rapid cell aggregation method using MC medium could agglutinate not only insoluble particulate matter but also fibre-like macromolecular proteins such as ECM components dispersed in the normal culture medium. Furthermore, by changing the ECM concentration, we could modulate the amount (thickness) of the ECM gel between cells (Fig. 1c–u). Subsequently, it is possible to vary the structure of fabricated spheroids in which cells are dispersed inside the ECM gel or are fully aggregated with a thin ECM layer between cells. Briefly, the aggregation method using MC medium allows the fabrication of various 3D microenvironments that are not limited by the type of ECM components.

In addition, our study demonstrated that temperature is another parameter modulating ECM gelation and the progression of aggregation. For example, the Matrigel used in this study gelates at 37 °C^33^. In fact, when cells and Matrigel were injected into MC medium under low-temperature conditions, the Matrigel was further condensed in spheroids because the gelation rate of ECM was slowed (Fig. 2 and see Supplementary Fig. S4). Taken together, our results indicate that it will be necessary to consider the ECM gelation parameters, such as the ECM concentration and temperature. Furthermore, MC medium has the potential to prevent the diffusion of water-dispersible macromolecular proteins and polysaccharides with a molecular weight of approximately 250,000 or higher (Fig. 3). However, the results also indicated that relatively small nutrients/proteins might be able to diffuse through the MC medium. For example, MC medium will not be able to prevent the diffusion of albumin (MW (molecular weight) = 66,000)^35^ as insulin (MW = 6,000)^36^ and cytokines (MW = 5,000–20,000)^37^. Efficient loading of protein molecules with molecular weights of approximately 40,000 into spheroids may be accomplished by binding them to macromolecules with molecular weights greater than 250,000. Specifically, macromolecular crowding occurs in MC medium^22^. For example, ECM components secreted from aggregated cells will remain nearby without diffusion in MC medium. Normally, the hepatic ECM comprises less than 3% of the relative area on a liver tissue section^38^. During chronic liver diseases, however, it is reported that amount of ECM is increased a 4 to 7 fold compared with that in normal liver^39^. When the amount of FITC-collagen in spheroids was quantified, the amount of 0.1 mg/ml FITC-collagen was increased approximately 7-folds compared with that of 0.2 mg/ml FITC-collagen (see Supplementary Fig. S1). Consequently, the aggregation method using MC medium will allow the fabrication of 3D tissues composed of cells and ECMs as a more physiological or a disease-mimicking model, which will be beneficial for tissue engineering, regenerative medicine, and pharmaceutical applications.

Cell encapsulation in biopolymer gels was initially investigated for immunoisolation of cells producing therapeutic proteins^40^. More recently, encapsulation of mammalian cells was expected to be applied for the regeneration of various tissues^41^. A popular method of generating ECM gel capsules is to prepare emulsions to form interfaces between water and oil. With the emulsion method, many capsules can be produced simultaneously, but some cells are damaged due to contact with oil. The encapsulation process resulted in cell death rates of approximately 20% and 30% in human fibroblasts and human umbilical vein endothelial cells, respectively^42^. Because encapsulation using MC medium was able to significantly prevent cytotoxicity (Fig. 4), it is considered to have great advantages compared to conventional methods.

In the tissue microenvironment, cell-cell and cell-ECM interactions affect cell physiology and function^43^. The difference in the amount of ECM mixed into the spheroids changed not only the cell aggregation state inside the spheroids but also hepatic function. Cells were separated from each other to inhibit cell-cell interactions in Matrigel capsules (Fig. 1i,n), and the albumin secretion activity was lower than that of non-ECM control spheroids (Fig. 5). Interestingly, when Matrigel was diluted, the cell-cell interaction was improved (Fig. 1p), and the albumin secretion activity at a Matrigel dilution of 30-fold relative to the original concentration was higher than that of the control spheroids (Fig. 5). Matrigel is isolated from Engelbreth-Holm-Swarm tumours developed in mice and consists of a mixture of laminin, type IV collagen, fibronectin, heparan sulfate, and many other components^33,44^. The composition of the ECM differs among organs and tissues *in vivo*, thereby affecting cell survival, morphology, shape, polarity, and differentiation^11^. The ECM thus allows cells to exhibit optimal functions for each organ and tissue. Therefore, the selection and combination of components and gelation control of the ECM are essential depending on the cell type.

Hepatocytes are polarized epithelial cells with distinct apical and basolateral domains^45,46^. The current *in vitro* gold standard, the ECM sandwich culture system proposed by Dunn *et al*.^47^, has been shown to be capable of developing and maintaining polarity in human and rat primary hepatocyte cultures^47–50^. Furthermore, it has also been reported that spheroid culture allows better development of hepatocyte polarity than sandwich culture^51^. In Hep G2 and HuH-7 spheroids, cell polarity formation was also observed in hanging drop culture and in ultra-low attachment plates^52–55^. However, these cell culture systems require at least six days to induce the formation of cell polarity in human hepatoma cells. In contrast, our method of culturing ECM-loaded spheroids with 0.3 mg/ml Matrigel facilitated the appearance of these features, such as localization of apical and basolateral markers, after culture for only two days (Fig. 7). Thus, these results indicate that Matrigel-loaded spheroids provide a better cell microenvironment for inducing hepatic polarity than conventional culture methods (spheroid culture by the hanging drop method, the ultra-low attachment plate method, and the 2D sandwich method).

Cell polarity and ECM signalling have been shown to promote liver-specific functions such as albumin secretion^56^. Matrigel-loaded spheroids induced higher albumin secretion activity than control spheroids (Fig. 5). This result suggests that the improvement in albumin secretion activity is caused by the development of cell polarity in spheroids. In our method, it was shown that Matrigel-loaded spheroids exhibited induction of cell polarity but minimal expression of metabolic enzymes, such as CYPs (Fig. 6). Another 3D culture system using a peptide nanofibre matrix was also shown to induce polarity in Hep G2 cells, although these spheroids did not exhibit upregulation of metabolic enzymes^57^, similar to our results.

Recently, the in-depth exploration of ECM composition, organization, and biological functions associated with the development of novel biocompatible materials has provided applications for various types of ECMs: tissue-derived ECMs, chemically synthesized polymers, and biomaterials derived from insects, such as silk from silkworms^58,59^. The aggregation method using MC medium to replenish those materials will be useful for *in vitro* generation of tissues that exhibit novel functions achieved by the interaction between cells and the replenished materials.

In conclusion, we present an aggregation method using MC medium that allows cell co-aggregation with water-soluble ECM components and macromolecular polysaccharides. Furthermore, by changing the ECM concentration, we could sequentially tune the amount of ECM gel between cells in spheroids in one step. Compared to conventional methods, the generation of ECM gel capsules in MC medium exerts a negligible influence on cell viability, in contrast to other capsulation methods such as oil emulsion. In addition, our method will be useful to establish microenvironments suitable for inducing liver-specific functions, such as albumin secretion activity and cell polarity, in 3D hepatic spheroid cultures.

## Methods

### Cell culture

Hep G2 cells, HuH-7cells, human liver vascular endothelial (TMNK-1) cells and human bile duct epithelial (MMNK-1) cells were obtained from the Japanese Center Research Bank and cultured in Dulbecco’s modified Eagle’s medium (DMEM; Wako) supplemented with 10% foetal bovine serum (Corning) and 100 units/ml of penicillin-streptomycin (Wako). Cells were grown in an incubator at 37 °C and supplied with 5% CO_2_. Mouse bone marrow cells were isolated from the femurs and tibias of 8-week-old C57BL/6NcrSlc male mice (Japan SLC) using previously described method^24^. Isolated cells were cultured in DMEM (containing 10% fetal bovine serum and 100 units/ml of penicillin-streptomycin) at 37 °C in a 5% CO_2_ incubator. All animal experiments conformed to the Guide for the Care and Use of Laboratory Animals and were approved by the Institutional Committees of Laboratory Animal Experimentation (Animal Research Center of Yokohama City University, Yokohama, Japan).

### Generation of spheroids containing cells and ECM components/macromolecular polysaccharides

We previously established a rapid aggregation system that allows cell aggregation cells by using 3% methylcellulose (MC; Sigma) medium^60^. MC medium was poured into a dish with a positive-displacement pipette (Gilson), because 3% MC medium is highly viscous. To visualize cell distribution by fluorescence in spheroids, Hep G2 cells were labelled with PKH26 (Sigma). Briefly, cells were suspended in 0.02 mM PKH26 solution in diluent C and incubated for five minutes at room temperature. The staining reactions were stopped by the addition of an equal volume of DMEM supplemented with 10% FBS, and cells were washed with phosphate-buffered saline (PBS). ECM components and macromolecular polysaccharides were mixed into the cell suspension, which included growth factor-reduced Matrigel (BD Biosciences), fluorescein isothiocyanate (FITC)-collagen (Chondrex), FITC-dextran (average molecular weights of 10,000, 40,000, and 250,000) (Sigma) and fibronectin (BD Biosciences)-labelled FITC by using a Fluorescein Labelling Kit–NH2 (Dojindo) according to the manufacturer’s instructions. ECM components were diluted at various ratios in normal culture medium. The cell density in suspension in the presence or absence of ECM components or macromolecular polysaccharides in normal culture medium (without MC) was adjusted to 2 × 10^6^ cells/ml or 1 × 10^6^ cells/ml. Spheroids composed of 2,000 cells or 1,000 cells were fabricated by injecting 1 μl of the abovementioned cell suspension into MC medium. Unless otherwise specified, injection of cell suspension to generate spheroids was carried out at room temperature. ImageJ (NIH) was used to quantify the amount of ECM per spheroid. It was calculated by dividing the area FITC signal of The ECM with the spheroid area. The aggregated spheroid or polysaccharide size was also evaluated by quantifying the spheroid area in each image by ImageJ. To quantify ECM thickness, spheroid images were obtained by using CQ1 confocal quantitative image cytometer (Yokogawa Electric).The images were analyzed by Cell pathfinder software (Yokogawa Electric).

### Haematoxylin and eosin staining

To isolate spheroids from MC medium, 0.5 volume of cellulose solution (Yakult Pharmaceutical Industry, adjusted to 5 U/ml with normal culture medium) relative to the volume of MC medium was added and incubated for 30 minutes at 37 °C to reduce the viscosity of the MC medium. Isolated spheroids were fixed with 4% paraformaldehyde (PFA, Wako) for 15 minutes at room temperature. Spheroids embedded in paraffin were sectioned at a thickness of 8 µm by a microtome. Sections were stained with haematoxylin (Wako) and eosin Y (Wako).

### Analysis of the dead cell rate

Aggregates consisting of Hep G2 cells and undiluted Matrigel were fabricated in 3% MC medium as described above or in mineral oil (Sigma) and were then incubated for 30 minutes. When mineral oil was used, cells suspended in undiluted Matrigel were injected into mineral oil by the same method as injection into 3% MC. ECM gel capsules treated with 4% PFA were prepared as the positive (100% dead) control. Next, ECM gel capsules were labelled with calcein-AM (Dojindo) and propidium iodide (Dojindo) according to the manufacturer’s instructions and were then evaluated by a Leica SP6 confocal laser microscope. The ratio of dead cell nuclear area was obtained by dividing the area of dead cell nuclei (red fluorescence) by the total area of those ECM gel capsules, as quantified by ImageJ. The resulting values were normalized to the mean value obtained from the positive controls to calculate the percentage of dead cells.

### Albumin secretion assay

After two days of culture in MC medium, Hep G2 and HuH-7 spheroids were isolated from MC medium as described above, transferred into 6-well plates and incubated for 24 hours. Conditioned medium was assayed for albumin concentration by using an albumin ELISA quantitation kit (Bethyl). In addition, the amount of genomic DNA in the spheroids was measured by a QuantiFluor dsDNA system (Promega) to normalize albumin secretion by cell number.

### Real-time PCR analysis

Spheroids composed of Hep G2 cells and HuH-7 cells were cultured in MC medium for two days. Total RNA was extracted using Trizol reagent (Invitrogen) and reverse transcribed into cDNA by using ReverTra Ace qPCR RT Master Mix (TOYOBO). Real-time PCR was performed with TaqMan gene expression master mix (Thermo Fisher) or SYBR green master mix (Bio-Rad). The target gene expression level was normalized to the GAPDH level. The primer and prove sequences are listed in Supplementary Table S1.

### Spheroid immunostaining

Paraffin sections of spheroids were deparaffinized and immersed in 10 mM citrate buffer (pH 6.0) (Wako) followed by autoclaving at 121 °C for 10 minutes. Sections were reacted with Primary antibody against zonula occludens-1 (ZO-1; Thermo Fisher) and stained with a Dako ENVISION Kit/HRP (DAB). Primary antibodies against Claudin-1 (Thermo Fisher), Ezrin (Santa Cruz), and Multidrug resistance-associated protein 2 (MRP2) (M2 III-6; Abcam) were incubated and stained with Alexa 488- or Alexa 568-conjugated goat anti-mouse or anti-rabbit IgG secondary antibodies and counterstained with Hoechst 33342 (Dojindo).

### Ultrastructural assessment

Spheroids composed of Hep G2 cells and HuH-7 cells were fixed with 3% glutaraldehyde and post-fixed with 1% osmium tetroxide. Fixed specimens were dehydrated and embedded in epoxy resin. Ultra-thin sections were observed under a transmission electron microscope (JEM-1400; JEOL).

### Statistical analysis

All values are expressed as the mean ± SEMs based on more than three samples per condition, and experiments were independently repeated at least twice. Statistical analyses were performed using Student’s t-test, one-way ANOVA with post hoc multiple comparisons by Tukey’s test procedure, or two-way ANOVA with post hoc multiple comparison by Sidak’s test procedure as specified in the figure legends, which were calculated by GraphPad Prism software. p  <  0.05 was considered as statistically significant.

## Supplementary information

Supplementary Information.
