# Supplementary material for: Development of a tunable method to generate various three-dimensional microstructures by replenishing macromolecules such as extracellular matrix components and polysaccharides
Source: Sci Rep. 2020 Apr 16;10:6567. doi: 10.1038/s41598-020-63621-4 (PMC7162899; doi:10.1038/s41598-020-63621-4)
Supplement: Supplementary file 1 — Supplementary Information. [file 41598_2020_63621_MOESM1_ESM.pdf]

Development of a tunable method to generate various three-dimensional microstructures by replenishing macromolecules such as extracellular matrix components and polysaccharides

Fumiya Tao[1], Kanae Sayo[1], Kazuyuki Sugimoto[2], Shigehisa Aoki[3], and Nobuhiko Kojima[1*]

[1]Graduate School of Nanobioscience, Yokohama City University, Yokohama, Japan

[2]Solution Division, Quality Assurance and Customer Support Center, Life Innovation Business Headquarters, Yokogawa Electric Corporation, Kanazawa, Japan

[3]Department of Pathology & Microbiology, Faculty of Medicine, Saga University, Saga, Japan

Corresponding author: Nobuhiko Kojima, Graduate School of Nanobioscience, Yokohama City University, 22-2 Seto, Kanazawa-ku, Yokohama 236-0027, Japan; Tel: +81-45-787-2214; Fax: +81-45-787-2413; E-mail: nobuhiko@yokohama-cu.ac.jp

Supplementary Information

Included Supplementary Material:

Supplementary Figures 1-5

Supplementary Table 1

a

FITC-collagen concentration	ECM thickness (μm) Mean	SE	P
0.2 mg/ml	1.264	0.174	-
0.1 mg/ml	0.857	0.192	<0.05
0.03 mg/ml	0.741	0.168	<0.05

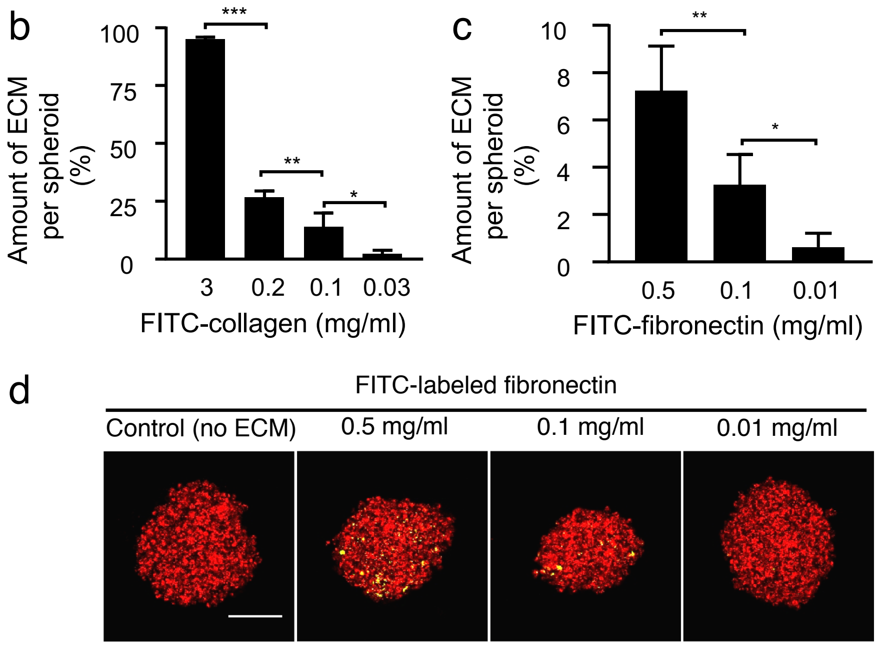

Supplementary Fig. S1. Quantification of the thickness amount of FITC-labeled ECM in spheroids.

The thickness of ECM around cells and amount of ECM per spheroid. (a) Quantification of the thickness of FITC-collagen around cells in Hep G2 spheroids. The data are the means ± SEMs, n=3-5. P value by one-way ANOVA vs. spheroids with 0.2 mg/ml FITC-collagen. (b) Quantification of the amount of FITC-collagen per Hep G2 spheroids. (c) Quantification of the amount of FITC-labelled fibronectin signals per HuH-7 spheroids. The data are the means ± SEMs, n=3-5. *: $p < 0.05$, **: $p < 0.01$, ***: $p < 0.001$ by one-way ANOVA. (d) ECM in the spheroids was visualized by using different dilution of FITC-labeled fibronectin (green) together with PKH26-stained HuH-7 cells (red). Bar = 100 μm.

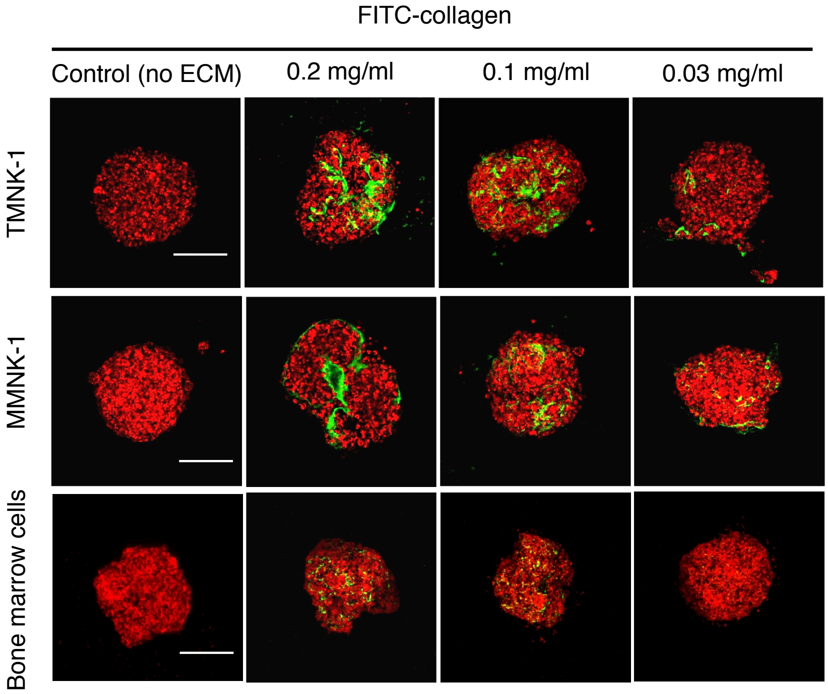

Supplementary Fig. S2. spheroid synthesis with variety cell types beyond liver carcinoma cell lines by using ECM.

ECM in the spheroids was visualized by using different dilutions of FITC-collagen (green) together with PKH26-stained TMNK-1 cells, MMNK-1 cells and bone marrow cells (red). Bar = 100 µm.

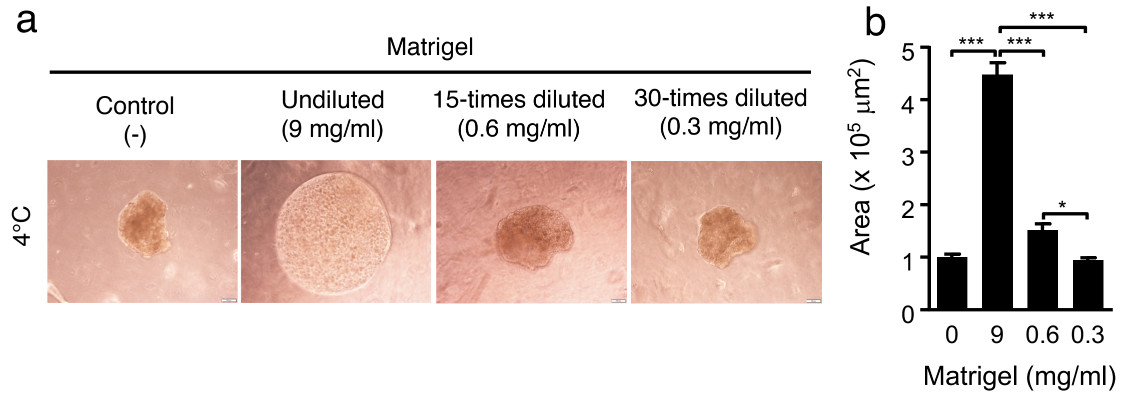

Supplementary Fig. S3. 3D structures responding to temperature.
Size of Hep G2 spheroids with and without Matrigel. All the procedures-mixing the cell suspension and Matrigel, injecting the suspension into MC medium, and incubating for 60 minutes-were performed at 4℃. (a). Those images were measured spheroid areas (b). The data are the means ±SEMs, n=7-10. *: $p < 0.05$, **: $p < 0.01$, ***: $p < 0.001$ by one-way ANOVA.

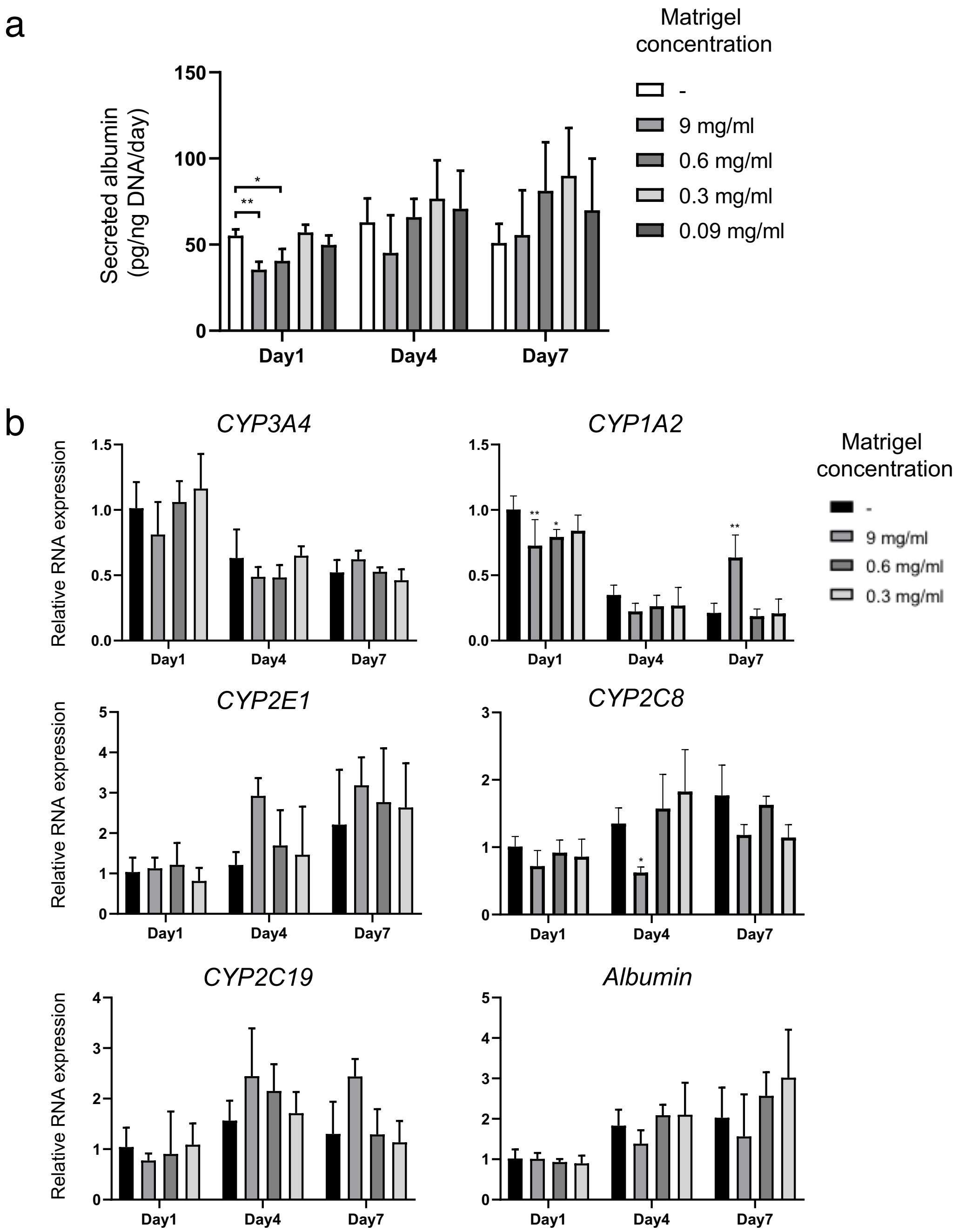

Supplementary Fig. S4. The albumin secretion and Liver-specific gene expression in Hep G2 spheroids in response to the ECM concentration on day1, day4 and day7.

(a) Secreted albumin levels in culture medium were measured by ELISA. In addition, spheroids incubated for albumin secretion assessment were extracted, and the amount of genomic DNA was measured to normalize albumin secretion by the number of cells. The data are the means ± SEMs, n=3. *: $p < 0.05$, **: $p < 0.01$ by one-way ANOVA. (b) Data are shown as the fold change against expression level in no-ECM control spheroid. The data represent the mean ± SEM, n=3. *: $p < 0.05$. **: $p < 0.01$ by one-way ANOVA vs. no-ECM control at each time point.

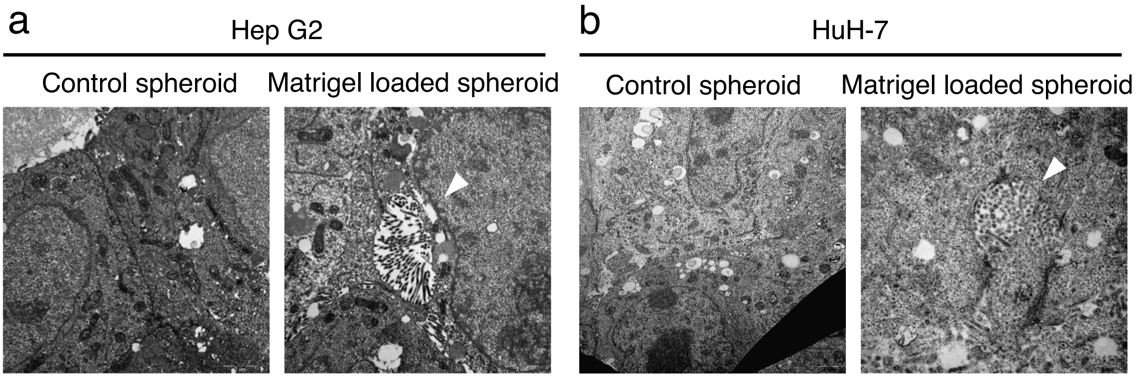

Supplementary Fig. S5. Observation of spheroid microstructure by electron microscope.
Electron microscopy images of Hep G2 (a) and HuH-7 (b) spheroids. Matrigel loaded spheroid was fabricated by using 0.3 mg/ml Matrigel. Bile canaliculus is indicated with white arrows.

TaqMan assay

Gene	Applied Biosystems TaqMan assay
CYP3A4	Hs00430021_m1
CYP1A2	Hs00167927_m1
GAPDH	Hs02758991_g1

SYBR Green assay

Gene	Forward primer	Reverse primer
CYP2E1	ACGGTATCACCGTGACTGTGG	GCATCTCTTGCCTATCCTTGA
CYP2C8	AGATCAGAATTTTCTCACCC	AACTTCGTGTAAGAGCAACA
CYP2C19	TTGAATGAAAACATCAGGATTG	GAGGGTTGTTGATGTCCATC
ALB	GCAAGGCTGACGATAAGGAG	CCTAAGGCAGCTTGACTTGC
GAPDH	GAGTCAACGGATTTGGTC	GGCAACAATATCCACTTTAC

Supplementary Table S1. Primers used for RT-PCR.
